# Body mass index and neuropsychological and emotional variables: joint contribution for the screening of sleep apnoea syndrome in obese

**DOI:** 10.5935/1984-0063.20200030

**Published:** 2021

**Authors:** Olga Rodrigues Ribeiro, Isabel do Carmo, Teresa Paiva, Maria Luísa Figueira

**Affiliations:** 1 Hospital Egas Moniz, Neuroscience - Lisbon - Portugal.; 2 ISAMB - Instituto de Saúde Ambiental - Lisbon - Portugal.; 3 Hospital de Santa Maria, Endocrinology, Diabetes and Metabolism - Lisbon - Portugal.; 4 Sleep Medicine Centre, Sleep Medicine - Lisbon - Portugal.; 5 Hospital de Santa Maria, Psychiatry Department - Lisbon - Portugal.

**Keywords:** Body Mass Index, Emotions, Cognition, Obesity, Morbid, Obstructive Sleep Apnea

## Abstract

**Objective:**

Obstructive sleep apnoea (OSA) is the most common sleep disorder and its prevalence has increased with the obesity pandemic. We aimed to explore the presence of OSA in severe obesity and to evaluate the association of body mass index (BMI) with sleep architecture, cognition, emotional distress and comorbidities in OSA versus non-OSA patients.

**Methods:**

A sample of 61 patients performed a neuropsychological battery that included tasks of attention, memory, perceptual/visuospatial ability, vocabulary, inhibition, cognitive flexibility and distress symptomatology, before overnight polysomnography.

**Results:**

More than half of the sample had OSA. Excessive daytime sleepiness was not a prominent complaint. Sleep architecture was worse in the OSA than in the non-OSA group, and hypertension was increased with OSA, especially in the severe OSA group. A higher BMI was associated with cognitive distress and sleep variables and with type 2 diabetes. The apnoea-hypopnoea index (AHI) was correlated with gender and cognitive measurements. Cognitive complaints were associated with enhanced distress in both OSA and non-OSA patients.

**Discussion:**

OSA is considerably present in severely obese patients. The BMI was strongly associated with other important anthropometric measurements along with worsening sleep architecture and lower executive functioning, both of which may contribute to weight gain. The AHI was significantly higher in men and affected memory and maintaining sets on the Wisconsin Card Sorting Test which may represent a barrier to treatment adherence for this disorder. The substantial presence of cognitive complaints in OSA and non-OSA patients suggests the need for psychological intervention focused on adaptive coping strategies, mostly for depressive symptoms. Given the current obesity epidemic, these results support the need for routine sleep investigation in obese people, particularly in primary care settings. BMI, neuropsychological and emotional screening can provide crucial information about asymptomatic and high-risk patients who require prompt sleep intervention and obesity treatment.

## INTRODUCTION

Obstructive sleep apnoea (OSA) is the most common sleep disorder. It is a chronic condition that has increased in prevalence over the past two decades. Its prevalence varies according to gender and age, and it is nearly twice as common in the obese population as in adults of normal weight^1^. OSA has a large economic impact, affects quality of life and is associated with work-related injuries. It is a major public health issue that is exponentially increasing owing to the increased prevalence of type 2 diabetes mellitus (DM) and obesity, ageing, and improvements in screening and testing methods^2^.

OSA is estimated to affect approximately 9% to 38% of the general population based on an apnoea-hypopnoea index (AHI) of ≥5 events/hour. It is more frequent in males and with advancing age^3^.

The condition is characterized by recurrent obstruction of the upper airway during sleep due to mechanical dysfunction of the throat’s musculature, resulting in a periodic cessation (apnoea) or reduction (hypopnoea) in breathing, with subsequent cyclic desaturations of arterial blood^4^. Consequences are impaired blood oxygenation, repetitive hypoxia, sympathetic nervous system activation, increased blood pressure, sleep fragmentation and excessive daytime sleepiness (EDS)^5^. It has a negative impact on the cardiovascular and metabolic systems and is highly associated with hypertension (HTN), DM and stroke^2^. Repetitive hypoxia has been considered a major contributor to cerebrovascular changes such as structural and functional deterioration that lead to cognitive deficits, particularly in attention, memory and executive functioning^6^.

OSA also affects mental health, with increased clinical levels of depressive symptoms, anxiety, irritability, fatigue or diminished energy resulting from the sleep deprivation and social effects of the illness^7^. Depression and anxiety symptoms, reduced activity levels, medication and increased appetite may contribute to weight gain, worsening OSA.

Considering the prevalence of obesity in patients with OSA, sufficient weight loss can confer benefits and significantly mitigate symptoms^1,8^. In fact, nighttime symptoms (snoring, gasping, insomnia or frequent arousals from sleep and morning headaches) and daytime symptoms (sleepiness, fatigue, poor concentration, irritability, depression, weight gain and decreased libido) should be confirmed by the medical evaluation and can be strengthened by the use of screening questionnaires and anthropometric measurements. Questionnaires and anthropometric measurements play an essential role in OSA recognition and should also be applied in primary care settings, focusing on asymptomatic patients and high-risk groups such as obese people^9,10^.

Body mass index (BMI) is the most common measurement of obesity. An increased BMI is considered a significant risk factor for early manifestations of neurodegenerative diseases and emotional distress, not only for depression or anxiety symptoms but also for a wider range of psychopathologies^11^. However, some authors have suggested that BMI is inferior to alternative measurements such as the waist-to-hip ratio (WHR) or waist-to-height ratio (WTHR), which are indicators of central obesity^(12,13.)^ Others have shown that these measurements are highly correlated with BMI^14^. They have a similar strength of association with risk of cardiovascular disease, although they have a lack of standardized measurement protocols, reference data and accuracy in severe obesity^15^.

The aims of our study were: (a) to explore the presence of OSA in a sample of severely obese patients seen at a Portuguese referral centre for the treatment of obesity; and (b) to evaluate the association of BMI, OSA and sleep architecture with cognitive functioning, emotional distress and comorbid diseases. This research is particularly important because: (1) it emphasizes the study of OSA based on a PSG for patients with extreme conditions such as severe obesity; (2) it highlights the importance of BMI as a screening tool for OSA; and (3) it stresses the association of BMI and AHI in OSA versus non-OSA patients, with the cognitive and emotional domains in a population less than 65 years of age (i.e., not yet considered elderly)^16^. Interventions may be more active in this age group. Additionally, the BMI constitutes an early and cost-effective screening method for patients at high risk of OSA, particularly in primary care settings, since it is in obese people that the incidence of OSA is higher^17^.

## MATERIAL AND METHODS

### Sample

The present study is part of a project presented to the Ethics Committee of the Centro Hospitalar Universitário de Lisboa Norte and was approved on March 2012. After obtaining approval from the Ethics Committee, we explored the results of neuropsychological and emotional evaluations of 61 patients with severe obesity (BMI≥40kg/m2) out of an initial number of 120, who sought treatment at the Morbid Obesity Consultation between May 2012 and December 2015, and were invited to perform overnight PSG at the Pulmonology Department. PSGs were performed with an *Alice* 5 device (Philips Respironics®, USA), included monitoring of respiratory flow, respiratory effort rate, arterial oxygen saturation, electroencephalogram and electromyogram of mentis muscles and were reviewed by a Pulmonologist with training in sleep disorders according to internationally agreed criteria from American Academy of Sleep Medicine 2007^18^.

We collected data related to sleep parameters like AHI, minimal oxygen saturation (MinSO2), mean oxygen saturation (MeanSO2), Rapid Eye Movement sleep (REM sleep), sleep latency, sleep efficiency, microarousals and non-REM stages N1, N2, N3, from the individual PSG record of nocturnal sleep.

Eligibility criteria for the study assessment were age between 18 and 65-years-old, without any known diagnosis of psychiatric or neurological disorders, had at least four years of schooling, had corrected hearing and vision and unknown OSA.

### Materials

We administered a sociodemographic/clinical questionnaire to collect personal characteristics and relevant clinical data. We gave patients a pen-paper neuropsychological battery of evaluation comprising Wechsler Intelligence for Adults (WAIS)-III^19^ [subtest of Digit Span (for attention and working memory), Digit Symbol (for fine motor control and learning speed), Search Symbol (for processing speed), Vocabulary (for level of education and acquired knowledge)], and Rey-Osterrieth Complex Rey Figure (RCF)^20^ for perceptual/visuospatial ability and memory; Rey Auditory Verbal Learning Test (RAVLT)^21^ for episodic memory, Stroop Colour Test^22^ for resistance to interference; Trail-making Test (TMT)^23^ for cognitive flexibility; Wisconsin Card Sorting Test (WCST)^24^ for problem solving and abstract thinking, and Hopkins Symptom Checklist-Revised (SCL-90-R, Portuguese Version)^25^ for distress symptomatology.

The selection of the neuropsychological battery was in line with the impact that obesity has on attention, perception, memory, executive functions and emotional performance of obese patients^11,26-28^. We sought to verify the impact of extreme obesity and OSA on cognitive function.

### Procedures

At the end of their required endocrinology consultation, patients were invited to participate in the study. We adopted a sequential sampling sequence, assuming a non-attendance rate of the first query superior to 30%^29^. We explained the purpose of the study to each patient, their voluntary interest was confirmed and informed consent information was obtained.

We took anthropometric measurements such as weight, height, neck circumference (NC), waist circumference (WC) and hip circumference (HC).

We calculated the BMI as the weight (kg) divided by the height squared (m2), the WHT as the WC (cm), divided by the HC (cm), and the WHTR as the WC (cm) divided by the height (cm).

We collected data from the clinical records of patients on vascular risk factors such as HTN, DM, dyslipidemia, snoring and cognitive complaints, (attention/concentration difficulties and poor mental flexibility) and we categorized them as a dichotomous variable: “present or not present”. We collect information about the number of hours of sleep per night on workdays and excessive daytime sleepiness (EDS) measured by the Epworth Sleepiness Scale values which scores varied from 0 (minimum) to 24 (maximum)^30^.

We considered 4 groups regarding the AHI: <5 (non-OSA), ≥5 (mild-OSA), between 15-30 (moderate-OSA) and ≥30 (severe-OSA)^31^.

Neuropsychological assessments were carried out individually, usually between 2pm and 6pm and prior to the performance of the PSG. They had an average duration of 45 minutes and was applied by a psychologist with a specialization focus in neuropsychology.

### Statistical analysis

The initial objective of the present work was to study the executive functioning through the WCST in severe obese patients with and without OSA, submitted to conventional versus surgical treatment for weight loss. Assuming that the WCST has a t-score of 50 and a standard deviation of 10, 50 patients per group would be needed to compare bariatric surgery with the conventional method for an alpha error of 5% and a power of 70%. Statistical analysis was performed using the Statistical Package for the Social Sciences version 24.0 for Windows software program (IBM Corp., Armonk, NY, USA).

Since most of the variables did not follow a normal distribution, we used non-parametric analytic methods: Spearman’s correlation test for quantitative measurements, Tau_b Kendall’s for correlations between quantitative and scaled measurements, Man-Whitney and Kruskal-Wallis tests to verify the homogeneity of distribution of variables by gender, age, qualifications and employment status and Chi-Square for dummy variables. The significance level was set at *p*<0.05.

## RESULTS

### Overall sample (n=61)

Sociodemographic and clinic characteristics are expressed in Table 1 and the anthropometric measurements as well as the difference of medians for genders are presented in Table 2.

**Table 1:** Sociodemographic and clinical characterization of the sample (n=61).

	n	%
Gender		
Female	49	80.3
Male	12	19.7
Age		
20-30 years	12	19.7
31-40 years	18	29.5
41-50 years	11	18.0
> 50 years	20	32.8
Marital status		
Singles	8	13.1
Married/unmarried couples	43	70.5
Separate/divorced	10	16.4
Employment Status		
Employed	31	50.8
Unemployed	22	36.1
Pensioners	6	9.8
Others	2	3.3
Qualifications		
1º Cycle of basic school	10	16.4
2º Cycle of basic school	9	14.8
3º Cycle of basic school	19	31.1
Secondary school	19	31.1
Bachelor/graduation/master	4	6.6
Income		
Without Income	5	8.2
< 500 euros	24	39.3
500-750 euros	18	29.5
750-1000 euros	7	11.5
1000-1500 euros	5	8.2
> 1500 euros	2	3.3
Clinical diagnosis		
Arterial hypertension	29	47.5
Type 2 diabetes	11	18.0
Dyslipidemia	6	9.8
Life habits		
Tobacco consumption	11	18.0
Alcoholic beverages consumption	8	13.1
Regular physical activity	13	21.3
Overweight since childhood	26	42.6
Complaints		
Cognitive complaints	20	32.8
Snoring	42	68.9
OSA Diagnosis		
Non-OSA	21	34.4
Mild-OSA	12	19.7
Moderate-OSA	10	16.4
Severe-OSA	18	29.5

**Table 2:** Descriptive statistics of the anthropometric measurements of the total sample and differences of medians for genders performed by *Man-Whitney test*.

	Total sample (*n*=61) Mean±SD	Men (*n*=12) Mean±SD	Women (*n*=49) Mean±SD	Differences of medians (*p* value)
Weight (Kg)	123.50±17.74	137.50±16.20	120.07±16.50	.004**
Height (cm)	162.52±9.41	172.66±11.89	160.04±6.81	.001**
Neck circumference (cm)	42.73±5.02	46.45±46.00	41.81±41.00	.000**
Waist circumference (cm)	128.04±14.37	138.75±13.23	125.42±13.50	.004**
Hip circumference (cm)	137±10.17	133.58±10.14	137.83±10.11	.211
Body mass index (Kg/m^2^)	46.71±5.95	46.18±4.01	46.84±6.36	.863
Waist-to-hip ratio (cm)	0.93± 10	1.04±.11	.91±.081	.000**
Waist-to-height ratio (cm)	.78±.09	.80±.091	.78±.095	.261

* *p*<0.05;

** *p*<0.01; SD = Standard deviation.

The age of the sample ranged between 20 and 63-years-old with a mean of 42.61±18 for women and 40.08±12.61 for men.

The distribution of the BMI, AHI and EDS by the severity of OSA together with the characteristics of the sleep parameters are shown in Table 3 and Table 4, respectively. Significant differences for the sociodemographic, sleep and anthropometric measurements between groups are expressed in Table 5.

**Table 3:** Descriptive statistics of body mass index, apnea hypopnoea index and Epworth Sleepiness Scale between OSA groups.

Measurements	Severity of Apnoea/Hypopnoea Syndrome			
	Non-OSA patients	Mild OSA patients	Moderate OSA patients	Severe OSA patients
	(*n* = 21)	(*n*=12)	(*n*=10)	(*n*=18)
	Mean±SD	Mean±SD	Mean±SD	Mean±SD
Body mass index (Kg/m^2^)	44.06±3.00	48.77±8.13	45.99±5.86	48.82±6.07
Apnoea/hypopnoea Index/hour	1.45±1.48	9.61±3.08	21.24±2.94	58.53±25.63
Epworth Sleepiness Scale (EDS)	5.05±3.73	6.00±4.20	7.90±3.72	7.22±5.07

SD=standard deviation.

**Table 4:** Descriptive statistics of sleep parameters of the total sample and Non-OSA and OSA patients.

Sleep parameters	Total sample	Non-OSA patients	OSA patients
	**(*n*=61)**	**(*n*=21)**	**(*n*=40)**
	**Mean±SD**	**Mean±SD**	**Mean±SD**
Apnoea/hypopnoea index (frequency/hour)	23.14±27.70	1.45±1.48	34.53±28.16
Mean oxygen saturation (%)	94.26±1.99	95.38±1.284	93.68±2.06
Minimal oxygen saturation (%)	81.60±9.40	88.33±6.11	81.60±9.40
REM sleep latency (minutes)	151.76±94.20	169.03±89.22	151.76±94.20
REM sleep (%)	12.56±6.07	13.41±6.47	12.12±5.88
Sleep efficiency (%)	80.08±13.69	82.59±11.47	78.76±14.69
Non-Rem N1 stage (%)	17.80±21.07	15.93±19.79	18.78±21.90
Non-Rem N2 stage (%)	65.69±9.94	66.04±8.69	65.50±10.64
Non-Rem N3 stage (%)	13.63±13.86	12.55±8.35	14.20±6.09
Arousal index (frequency/hour)	32.78±24.67	20.28±11.42	39.34±27.22
Excessive daytime sleepiness	6.34±4.301	5.05±3.735	7.03±4.463
Number hours slept by night	7.163±1.104	7.245±.952	7.120±1.186

SD=standard deviation.

**Table 5:** Significant differences for the sociodemographic, sleep and anthropometric measurements between groups.

Variables	Groups comparisons (Man-Whitney Z and p value)					
	1 and 2	1 and 3	1 and 4	2 and 3	2 and 4	3 and 4
Age			-.2.817			
			.006*			
Apnea/Hypopnea Index	-4.721	-4.42	-5.328			
	.000**	.000**	.000**			
Mean Oxygen Saturation			-3.540			
			.000**			
Minimal Oxygen Saturation	-2.774	-2.858	-4.119			
	.005**	.003**	.000**			
Microarousals			-3.945			
			.000**			
REM sleep latency		-2.282			-2.117	-2.493
		.022*			.034*	.013*
Weight			-2.635			
			.007**			
Neck circumference			-4.409	-2.702	-3.489	
			.000**	.006**	.000**	
Waist circumference			-2.963			
			.003**			
Body Mass Index			-2.564			
			.010*			
Waist-to-hip ratio			-2.014			
			.043*			
Waist-to-height ratio			-2.578			
			.010*			

* *p*<0.05;

** *p*< 0.01; Group 1: non-OSA; Group 2: mild-OSA; Group 3: moderate-OSA; Group 4: severe-OSA.

The unemployed patients revealed a greater BMI (*K-W*=10.355, *p*=.016) than the employed or pensioners patients.

BMI was strongly correlated with NC (*r*=.405, *p*=.001), WC (*r*=.327, *p*=.010), HC (*r*=.705, *p*=.000), WHT (*r*=.293, *p*=.022) and WHTR (*r*=.789, *p*=.000).

OSA was presented in 65.6% of the sample (*n*=40), 80% were women (*n*=32), 20% men (*n*=8) with the majority reporting to snore (72.5%, *n*=29) and to have no cognitive complaints (67.5%, *n*=27).

AHI significantly increased with weight (*r*=.379, *p*=.003), NC (*r*=.608, *p*=.000), WC (*r*=.437, *p*=.000), BMI (*r*=.354, *p*=.005), WHR (*r*=.327, *p*=.010) and WHTR (*r*=.371, *p*=.003) and over age groups (*K-W*=9.002; *p*=.029).

HTN was the only diagnosed comorbidity that showed to be significantly higher in OSA patients than in non-OSA patients (*χ*2=10.659, *p*=.014; non- OSA=13,8%; mild-OSA=24.1%; moderate-OSA=20.7%; and severe- OSA=41.4%).

### Non-OSA versus OSA patients

The mean age of the non-OSA patients (*n*=21; µ=36.10±12.165) was lower than the mean age of the OSA patients (*n*=40; µ=45.28±9.743) and significantly different for the non-OSA group and the severe OSA group (*U*=-2.817; *p*=.006). HTN was considerably present in the OSA patients (χ2=10.659, *p*=.014; non-OSA with HTN=13.8% versus OSA with AHT=86.2%).

BMI was highly associated with the AHI when we compared the non-OSA group and the group including all the patients with OSA (*r*=.354, *p*=.005).

Significant associations between the BMI, AHI and cognitive, emotional and comorbid variables are presented in Table 6.

**Table 6:** Significant nonparametric correlations (Tau_b Kendall and Spearman) between body mass index and apnoea/hypopnoea Index and sociodemographic, cognitive, emotional, sleep and comorbid variables for Non-OSA patients (n=21) and OSA patients (n=40).

		Non-OSA patients		OSA patients	
		(*n*=21)		(*n*=40)	
Variables	Items	BMI	AHI	BMI	AHI
Sociodemographic characteristics	Gender		.411*		.430**
	Income	-.449*			.268*
Sleep measurements	Mean Oxygen Saturation			-.483**	
	Minimal Oxygen Saturation			-.525**	
Cognitive measurements	Deferred Recognition				-.354*
	Stroop Interference			-.392*	.383*
	Rey Complex Figure (Copy)			-.317*	
	WCST administered trails			.404**	
	WCST % errors			.315*	
	WCST % perseverative responses			.318*	
	WCST % concept. level responses			-.329*	
	WCST nr. completed categories			-.423**	
	WCST failure to maintain set				.315*
	WCST learning to learn	-.554**			
Emotional measurements	Global Severity Index			.318*	
	Phobic anxiety			.416**	
Comorbidities	Diabetes			.389**	

* *p*< 0.05;

** *p*<0.01.

Cognitive complaints for the non-OSA patients were associated with obsessive-compulsive symptoms (*r*=.432, *p*=.023), interpersonal sensitivity (*r*=.468, *p*=.014), phobic anxiety (*r*=.462, *p*=.019) and psychoticism (*r*=.482, *p*=.013).

Cognitive complaints for the OSA patients were considerable associated to symptom distress index (*r*=.308, *p*=.020), somatization (*r*=.314, *p*=.019), interpersonal sensitivity (*r*=.277, *p*=.040), depression (*r*=.373, *p*=.005), anxiety (*r*=.276, *p*=.041), phobic anxiety (*r*=.294, *p*=.033) and psychoticism (*r*=.275, *p*=.045).

## DISCUSSION

More than half of our sample was revealed to have OSA and an excess of generalized and central adiposity, reflected by anthropometric measurements that were significantly above those established for severe obesity. The women were slightly older than the men, who presented the highest weights greatest neck and waist circumferences and greatest local adiposity. The high neck and waist circumferences and severest OSA in men support the need for routine sleep investigation in obese people because the pattern of fat distribution and ageing is described as related to increased morbidity and mortality, generally associated with OSA^32^.

EDS is considered a clinically important symptom of OSA; this value was well above the average value of 4.6 of the general population^33^. Nevertheless, it was not related to the AHI and did not appear as a cardinal symptom. Similarly, a recent Portuguese study performed in a sleep clinic evaluated the perception of sleepiness before OSA treatment. The perception of EDS prior to treatment significantly increased after treatment because it was underestimated by the patients^30^. Kapur et al.^34^ also found no association between EDS and the AHI in a cohort study of 6,440 subjects. Specialists have argued the necessity for specific tools to evaluate the risk of OSA because screening questionnaires such as the Epworth Sleepiness Scale have very weak sensitivity and are not good predictors of the disorder^35^. The absence of variation between the OSA groups in the number of hours slept per night may have contributed to the lack of variation in the EDS. Collecting data from a population with a normal BMI for comparison could be useful to further investigate this finding.

The BMI was strongly related to oxygen desaturation and DM in OSA patients. Previous studies have shown that long-term protracted intermittent hypoxia can result in sleep fragmentation, cerebral vascular deficits, cognitive impairments and neurodegeneration, possibly due to cumulative effects^36^. This exposure leads to altered insulin resistance and glucose disposal, impacting the incidence of DM, which should motivate the early identification and intervention of OSA^37^. Intermittent hypoxemia has also been proposed to influence mood among OSA patients and suggests the need for interventions focused on the enhancement of adaptive coping strategies, particularly for dealing with depressive symptoms^38^. These interventions may help to optimize the standard treatment of OSA patients and could contribute to reducing psychological distress^39^.

Our investigation highlights the association between the BMI and the global severity index of distress symptomatology and phobic anxiety in OSA patients. An important aspect seems to be the general dimension of symptoms of maladjustment that also encompass social anxiety and agoraphobic symptoms, with consequent decreases in social contact, social activities and occupational activities, reinforcing weight gain and possibly OSA^40^. The patient symptoms revealed that significant emotional discomfort is associated not only with OSA but also with the degree of obesity. The symptoms may be linked to the lower activity levels and increased passive coping strategies reported by OSA patients, but these are also referred to by severely obese patients^41^.

Our investigation also stressed that in addition to the presence of emotional symptomatology, the increased BMI was linked to a decrease in the core abilities of executive functioning in OSA patients, which has also been described in the obese population^42^. Results of the Stroop test interference, WCST and RCF in OSA patients emphasize the reduction of inhibitory control, cognitive flexibility, problem solving, planning and perceptual/sensorial understanding of the environment, suggesting a dysfunctional executive profile. Inhibitory control refers to the general ability to withhold or inhibit inappropriate behaviour and its decline has been implicated in a range of impulsive psychiatric disorders that have high comorbidity, along with maladaptive eating behaviours and obesity. Inefficient inhibitory control is related to the inability to maintain consistent directed attention over time and may therefore play a role in the development and/or maintenance of obesity^43^. Visuoperceptive and cognitive flexibility difficulties suggest a decline in the ability to organize, plan and solve problems, which can result in additional problems with generating alternative behaviour patterns when presented with ambiguous information and adjusting behaviour when the rules change, namely, in dealing with food cues. Reduced executive functioning has been systematically reported and may represent a barrier to adherence to new nutritional guidelines and a tendency to continue the previous regime, perpetuating weight gain, obesity and possibly OSA^44^. Enhancing the information offered by healthcare providers and traditional sources, furnishing lifestyle change interventions, empowering patient with adequate eating habits, provide cognitive therapy behavioral, support groups and cognitive training are all important for a complementary approach to the patient^45^. These tools, however, should not delay the provision of bariatric surgery, the gold standard treatment for severe obesity and a valuable treatment option for OSA, regardless of the specific type^32,46^.

In contrast, the AHI is not associated with the interference of Stroop performance but rather with difficulties in episodic memory, especially in deferred recognition, which may indicate the failure to use adequate strategies to manipulate and organize verbal information. Alterations in hippocampal structures have been reported in patients with OSA. This cerebral structure is extremely sensitive to hypoxic damage, and its atrophy is associated with memory impairments^47^. A reduced recognition may be responsible for the increased difficulties in maintaining sets on the WCST, rather than a disturbance in selective attention, which could also influence the interference task. Increased difficulties in response maintenance may lead these patients to higher vulnerability situations, such as when trying to sustain a consistent adherence to the CPAP treatment.

Concerning the architecture of sleep, most patients with OSA present the severest impairments. These patients typically show sleep latency greater than 30 minutes, a severe microarousal index over 10, sleep efficiency less than 85% and a mean MinSO2 of less than 85%, indicating desaturation of oxyhemoglobin, which is associated with significant sleep fragmentation and inefficiency. These characteristics are similar to the findings from an Australian sample and are consistent with pathophysiological mechanisms linking OSA to DM and a decline in oxygen saturation^48^. The current study shows the importance of measurements such as BMI as markers for the diagnosis of DM in OSA patients, a relationship that was very clear in our sample.

Notably, most of the patients mentioned that the onset of obesity was in childhood. Previous reports have found that the higher prevalence of OSA is not confined to adults, with obese children recently showing a 46% prevalence of OSA compared to 33% of children seen in a general paediatric clinic^1^. This may indicate the importance of a prompt diagnosis of OSA and the prevention of early obesity owing to their ability to increase the risk for pathologies such as DM, HTN, dyslipidemia, or neurocognitive impairment.

HTN was significantly increased with the severity of OSA, indicating a clear relationship. We hypothesized that a larger sample size would emphasize other comorbidities that are associated with OSA, such as dyslipidemia.

We concluded that BMI was far above average in the sample, representing a valuable marker for ‘health risk’^49^. The BMI increased with the severity of OSA, although it showed no significant relationship with AHI. Moderate OSA was more strongly associated with lower BMI than mild OSA was, meaning that moderate OSA may be worse but less symptomatic, which requires further study.

Limitations regarding the representativeness of the studied population include our small sample size, the disproportion of men and women, and the absence of a healthy control group. The sample size may explain the association of the BMI and neuropsychological performance only with the OSA patients. We conjectured that with a large sample this association would include also the non-OSA patients. However, it is important to refer that our predominantly female sample is similar to the typical candidates for treatment of severe obesity in Portugal, and we compared patients with and without OSA^50^. A strength of this study is that PSG was used as the standard test for diagnosing OSA. This methodology reinforced that the higher the BMI, the greater the severity of OSA; therefore, BMI is particularly relevant to this sleep disorder. Because of their significance, the results should be further investigated with a large number of patients and after CPAP treatment.

## CONCLUSION

In summary, we have shown that OSA is significantly present in severely obese patients. BMI is well correlated with cognitive measurements, sleep, emotional parameters and comorbidities in OSA patients. The BMI is objective, cheap and easy to obtain. It can be an important first step and a good start in identifying asymptomatic patients to categorize populations at high risk of OSA, especially in primary care settings.

Particular attention must be given to distress symptomatology. This seems to be associated with cognitive complaints in OSA and non-OSA patients, suggesting a need for psychological interventions focused on adaptive coping strategies that deal with depressive symptoms. Neuropsychological screening may provide information about cognitive impairment, mostly associated with executive functioning, which may constitute a barrier to the adherence to treatment guidelines, perpetuating obesity and OSA.

These findings may offer additional insight into the management of obese patients outside of sleep units who require follow-up, because these units are highly specialized and have long patient waitlists and scarce human resources.

## References

[1] Romero-Corral A, Caples SM, Lopez-Jimenez F, Somers VK (2010). Interactions between obesity and obstructive sleep apnea: implications for treatment. Chest.

[2] Barewal RM (2019). Obstructive sleep apnea: the role of gender in prevalence, symptoms, and treatment success. Dent Clin North Am.

[3] Ornelas C, Carreiro A, Domingos A, Reis R, Frias L, Pavão C (2019). Relação entre doenças pulmonares obstrutivas e síndrome de apneia obstrutiva do sono. Rev Port Imunoalergologia.

[4] Wu PH, Rodríguez-Soto AE, Rodgers ZB, Englund EK, Wiemken A, Langham MC (2019). MRI evaluation of cerebrovascular reactivity in obstructive sleep apnea. J Cereb Blood Flow Metab.

[5] Przybylska-Kuc S, Zakrzewski M, Dybala A, Kicinski P, Dzida G, Myslinski W (2019). Obstructive sleep apnea may increase the risk of Alzheimer's disease. PLoS One.

[6] Alex RM, Mousavi ND, Zhang R, Robert J, Behbehani GK (2017). Obstructive sleep apnea: brain hemodynamics, structure, and function. J Appl Biobehav Res.

[7] Bardwell WA, Berry CC, Ancoli-Israel S, Dimsdale JE (1999). Psychological correlates of sleep apnea. J Psychosom Res.

[8] Hilsendager CA, Zhang D, McRae C, Aloia M (2016). Assessing the influence of obesity on longitudinal executive functioning performance in patients with obstructive sleep apnea syndrome. Obes Res Clin Pract..

[9] Steinberg S, Louis M, Salam SO (2019). Medical evaluation of patients with obstructive sleep apnea. Modern management of obstructive sleep apnea.

[10] Pasha S (2019). Screening for obstructive sleep apnea: should we do it?. Curr Pulmonol Rep..

[11] Ribeiro O, Carmo I, Paiva T, Figueira ML (2020). Neuropsychological profile, cognitive reserve and emotional distress in a portuguese sample of severely obese patients. Acta Med Port.

[12] Hartanto A, Yong JC (2018). Measurement matters: higher waist-to-hip ratio but not body mass index is associated with deficits in executive functions and episodic memory. Peer J.

[13] Nevill AM, Stewart AD, Olds T, Duncan MJ (2018). A new waist-to-height ratio predicts abdominal adiposity in adults. Res Sport Med.

[14] Ashwell M, Gibson S (2016). Waist-to-height ratio as an indicator of early health risk: Simpler and more predictive than using a matrix based on BMI and waist circumference. BMJ Open.

[15] Adab P, Pallan M, Whincup PH (2018). Is BMI the best measure of obesity?. BMJ.

[16] Ministério da Saúde (PT) (2017). Estratégia nacional para o envelhecimento ativo e saudável 2017-2025.

[17] Ribeiro JP, Araújo A, Vieira C, Vasconcelos F, Pinto PM, Seixas B (2020). Undiagnosed risk of obstructive sleep apnea in obese individuals in a primary health care context. Acta Med Port.

[18] Sateia MJ (2014). International classification of sleep disorders: third edition: highlights and modifications. Chest.

[19] Weschsler D (2008). Escala de inteligência de Wechsler para adultos.

[20] Rey A (1959). Teste de cópia de figuras complexas.

[21] Cavaco S, Gonçalves A, Pinto C, Almeida E, Gomes F, Moreira I (2015). Auditory verbal learning test in a large nonclinical Portuguese population. Appl Neuropsychol Adult.

[22] Fernandes S (2013). Stroop: teste de cores e palavras: manual.

[23] Cavaco S, Pinto C, Gonçalves A, Gomes F, Pereira A, Malaquias C (2008). Trail making test: dados normativos dos 21 aos 65 anos. Psychologica.

[24] Heaton R, Chelune G, Talley J, Kay G, Curtiss G (2005). Teste Wisconsin de classificação de cartas. Manual revisado e ampliado.

[25] Baptista A (1993). A génese da perturbação de pânico: a importância dos factores familiares e ambientais durante a infância e a adolescência.

[26] Sousa S, Ribeiro O, Horácio J, Faísca L (2012). Funções executivas em sujeitos candidatos e submetidos a cirurgia bariátrica. Psic Saúde Doenças.

[27] Ribeiro O, Grencho D, Carmo I, Paiva T, Figueira L, Horácio G (2015). Characterization of executive functioning in a Portuguese sample of candidates for bariatric surgery. Psychol Community Heal.

[28] Cohen RA (2010). Obesity-associated cognitive decline: excess weight affects more than the waistline. Neuroepidemiology.

[29] Camolas J, Santos O, Mascarenhas M, Moreira P, Carmo I (2015). INDIVÍDUO: intervenção nutricional direcionada aos estilos de vida em indivíduos com obesidade. Acta Port Nutr.

[30] Guimarães C, Martins MV, Rodrigues LV, Teixeira F, Santos JM (2012). Escala de sonolência de Epworth na síndroma de apneia obstrutiva do sono: uma subjetividade subestimada. Rev Port Pneumol..

[31] Thorpy M, Chokroverty S (2017). International classification of sleep disorders. Sleep disorders medicine.

[32] Peromaa-Haavisto P, Tuomilehto H, Kössi J, Virtanen J, Luostarinen M, Pihlajamäki J (2016). Prevalence of obstructive sleep apnoea among patients admitted for bariatric surgery: a prospective multicentre trial. Obes Surg.

[33] Bilyukov RG, Nikolov MS, Pencheva VP, Petrova DS, Georgiev OB, Mondeshki TL (2018). Cognitive impairment and affective disorders in patients with obstructive sleep apnea syndrome. Front Psychiatry.

[34] Kapur VK, Auckley DH, Chowdhuri S, Kuhlmann DC, Mehra R, Ramar K (2017). Clinical practice guideline OSA American Academy of Sleep Medicine clinical practice guideline. J Clin Sleep Med.

[35] Mayos M, Peñacoba P, Pijoan AMP, Santiveri C, Flor X, Juvanteny J (2019). Coordinated program between primary care and sleep unit for the management of obstructive sleep apnea. NPJ Prim Care Respir Med.

[36] Devita M, Montemurro S, Ramponi S, Marvisi M, Vilani D, Raimondi MC (2017). Obstructive sleep apnea and its controversial effects on cognition. J Clin Exp Neuropsychol.

[37] Nagayoshi M, Punjabi NM, Selvin E, Pankow JS, Shahar E, Iso H (2016). Obstructive sleep apnea and incident type 2 diabetes. Sleep Med.

[38] BaHammam AS, Kendzerska T, Gupta R, Ramasubramanian C, Neubauer DN, Narasimhan M (2015). Comorbid depression in obstructive sleep apnea: an under-recognized association. Sleep Breath.

[39] Timkova V, Nagyova I, Reijneveld SA, Tkacova R, Van Dijk JP, Bültmann U (2018). Psychological distress in patients with obstructive sleep apnoea: the role of hostility and coping self-efficacy. J Health Psychol..

[40] Brunault P, Jacobi D, Miknius V, Bourbao-Tournois C, Huten N, Gaillard P (2012). High preoperative depression, phobic anxiety, and binge eating scores and low medium-term weight loss in sleeve gastrectomy obese patients: a preliminary cohort study. Psychosomatics.

[41] Bardwell WA, Ancoli-Israel S, Dimsdale JE (2001). Types of coping strategies are associated with increased depressive symptoms in patients with obstructive sleep apnea. Sleep.

[42] Gunstad J, Paul RH, Cohen RA, Tate DF, Spitznagel MB, Gordon E (2007). Elevated body mass index is associated with executive dysfunction in otherwise healthy adults. Compr Psychiatry.

[43] Bartholdy S, Dalton B, O'Daly OG, Campbell IC, Schmidt U (2016). A systematic review of the relationship between eating, weight and inhibitory control using the stop signal task. Neurosci Biobehav Rev.

[44] Gameiro F, Perea MV, Ladera V, Rosa B, García R (2017). Executive functioning in obese individuals waiting for clinical treatment. Psicothema.

[45] Camolas J (2020). Empowering patients with adequate eating habits in the context of bariatric surgery. Acta Med Port.

[46] Sarkhosh K, Switzer NJ, El-Hadi M, Birch DW, Shi X, Karmali S (2013). The impact of bariatric surgery on obstructive sleep apnea: a systematic review. Obes Surg.

[47] Canessa N, Castronovo V, Cappa SF, Aloia MS, Marelli S, Falini A (2011). Obstructive sleep apnea: brain structural changes and neurocognitive function before and after treatment. Am J Respir Crit Care Med.

[48] Appleton SL, Vakulin A, McEvoy RD, Wittert GA, Martin SA, Grant JF (2015). Nocturnal hypoxemia and severe obstructive sleep apnea are associated with incident type 2 diabetes in a population cohort of men. J Clin Sleep Med.

[49] Unal Y, Ozturk DA, Tosun K, Kutlu G (2019). Association between obstructive sleep apnea syndrome and waist-to-height ratio. Sleep Breath.

[50] Gaio V, Antunes L, Namorado S, Barreto M, Gil A, Kyslaya I (2018). Prevalence of overweight and obesity in Portugal: results from the First Portuguese Health Examination Survey (INSEF 2015). Obes Res Clin Pract..

